# FEASIBILITY OF A DIGITAL FALL PREVENTION EXERCISE PROGRAM FOR AT-RISK OLDER ADULTS

**DOI:** 10.1093/geroni/igad104.1262

**Published:** 2023-12-21

**Authors:** Nicole Bajdek, Nancy Latham, Mary Dishaw, Shannon Farrell, Alisha Williams, Naomi Hachen, Kieran Reid

**Affiliations:** Brigham and Women’s Hospital, Boston, Massachusetts, United States; Brigham and Women’s Hospital, Boston, Massachusetts, United States; Brigham and Women’s Hospital, Boston, Massachusetts, United States; Brigham and Women’s Hospital, Boston, Massachusetts, United States; Best Buy Health, Inc., Richfield, Minnesota, United States; Best Buy Health, Inc., Richfield, Minnesota, United States; Brigham and Women’s Hospital/Harvard Medical School, Boston, Massachusetts, United States

## Abstract

Falls have serious consequences and are a leading cause of disability, institutionalization, and mortality for older adults. While exercise programs with in-person supervision reduce the occurrence and risk of falls, there are accessibility and adherence barriers associated with older adults’ participation in such programs. Digitally delivered home-based exercise interventions utilizing wearable technology and mobile applications may be an alternative fall prevention strategy for many vulnerable older adults. We evaluated the feasibility of a progressive 12-week, digitally delivered fall prevention exercise program in 23 at-risk older adults who reported ≥ 2 falls or ≥ 1 injurious fall in the past year, or a fear of falling (mean age: 76.6±6 yrs; BMI: 28.9±9 kg/m2; baseline short physical performance battery (SPPB) score: 8.9±3; 73.9% females). The structured exercise program was delivered via a tablet device and consisted of moderate-intensity strength and balance training with an embedded educational component (3x/wk) and walking exercise. A study interventionist provided participants with regular motivational coaching telephone calls throughout the program. Overall program adherence rates were high (≥ 80%) with minimal safety concerns. Preliminary efficacy findings revealed trends for improvements in fear of falling, cognitive performance, gait speed and overall SPPB score. The present study shows that a digitally delivered, home-based exercise program is feasible for older adults with elevated fall risk. Additional studies are warranted to confirm the efficacy of this highly scalable fall prevention strategy which could have important benefits for large populations of at-risk older adults.

